# Social Norms Influencing the Local Food Environment as Perceived by Residents and Food Traders: The Heart Healthy Hoods Project

**DOI:** 10.3390/ijerph16030502

**Published:** 2019-02-11

**Authors:** María Sandín Vázquez, Jesús Rivera, Paloma Conde, Marta Gutiérrez, Julia Díez, Joel Gittelsohn, Manuel Franco

**Affiliations:** 1 Surgery and Medical and Social Sciences Department, School of Medicine, University of Alcalá, Alcalá de Henares, 28871 Madrid, Spain; manuel.franco@uah.es; 2 Social and Cardiovascular Epidemiology Research Group, School of Medicine, Universidad de Alcalá, Alcalá de Henares, 28871 Madrid, Spain; jrivera@usal.es (J.R.); p.conde@uah.es (P.C.); julia.diez@uah.es (J.D.); 3 Sociology and Communication Department, Social Sciences Faculty, Salamanca University, 37008 Salamanca, Spain; magusa@usal.es; 4 Center for Human Nutrition and Global Obesity Prevention Center (GOPC) at Johns Hopkins University, Baltimore, MD 21205, USA; jgittel1@jhu.edu; 5 Department of Epidemiology, Johns Hopkins Bloomberg School of Public Health, Johns Hopkins University, Baltimore, MD 21205, USA

**Keywords:** food environment, food choice, qualitative methodology, neighborhoods

## Abstract

Exploring subjective elements of the food environment remains key to understand why and how residents purchase food. Our aim was to explore and describe the social norms relating to the local food environment and food purchasing behaviors, as perceived by residents and food traders in Madrid, Spain. This qualitative study took place in a middle socioeconomic status neighborhood of Madrid between January 2015 and May 2016. We conducted 35 semi-structured interviews. We used stratified purposive sampling to recruit residents, neighborhood workers (*N* = 20) and food traders (*N* = 15) representing different levels of involvement with food purchasing behaviors. We analyzed these data using an interpretative phenomenological analysis approach. Participants highlighted social aspects of the food environment in relation to food purchasing behaviors. First, interpersonal and relational food environment elements were emphasized, including trust and tradition. Participants also identified generational demographic trends in relation to changes in the way residents purchased food: the new pace of life and the lack of time to buy fresh food and to cook at home. All these elements were influenced by the economic crisis. Food environment interventions aiming to improve food purchasing behaviors and residents’ diets should consider intermediate social aspects of the food environment like trust and tradition and the fast pace of life of younger generations.

## 1. Introduction

A growing body of literature suggests that the local food environment has an influence on household and individual diets [1,2]. The local food environment can be defined as all aspects of the physical and social environment that influence individual dietary choices and behaviors [3,4]. To better understand the impact of environmental influences on dietary behaviors, it is important to capture people’s perceptions of these aspects [4].

Dietary behaviors are related to different physical and social environmental factors, such as the availability of healthy foods, the affordability of healthier food options, the nutritional adequacy of available foods, transportation and distributions systems, the cultural appropriateness of the food supply, and social norms [5]. Healthy diet improvements are more likely to occur and to be sustained if the environment supports healthy food options [6,7].

We argue that the context of the food environment is broad and that it needs to be studied holistically [8]. One’s diet is not only comprised of nutrients but also carries additional meaning where many existing elements and relationships of society are reproduced [9]. In this sense, the concept “habitus” (a group of generative schemes on the basis of which subjects receive and act in the world) [10] ties in with the idea of diet as a habit. Moreover, these social meanings may help to account for the differences in dietary practices between social groups, as well as between certain collectives (e.g., immigrants). Worth highlighting are studies using qualitative methodologies, which may enable a deeper exploration of aspects that together define neighborhood life, such as cultural influences, values, and social norms. These, in turn, affect dietary behaviors and thereby health [11,12].

A large number of studies have shown the influence of the local food environment on diet. What these studies have in common is their use of quantitative methods to measure specific dimensions of the food environment and to then observe how these relate to dietary behaviors [13,14,15,16,17]. Researchers have argued that there are unique features of Mediterranean food environments, which can influence healthy food accessibility [18]. In settings where the Mediterranean diet is consumed, food environments differ in terms of their diversity of the types of stores and their compactness in their urban form, which can help promote spatial access to healthy food [18]. As such, small retailers remain very prevalent [19] and the number of outlets per resident is three times higher (as compared to countries like the UK or the USA) [20]. In Spain, we have found associations between neighborhood social and economic changes from 2009 to 2013 and changes in the retail food environment from 2013 to 2017 in Madrid [19]. Compared to aging areas, new housing/gentrifying areas (with increasing socioeconomic status) showed a higher presence and proportion of supermarkets, compared to specialized stores (e.g., fruit and vegetable stores).

A key strategy for understanding the social dimensions of the food environment is the use of qualitative research. Qualitative methodologies and approaches can be used to document human behavior within social and cultural contexts and in this case will allow community members to articulate their experience within the food environment in their own words and describe the social norms associated with this experience [21]. Social norms are informal understandings that govern the behavior of certain groups in a given society [22] that can be considered intermediate-level factors between individual behaviors and macrosocial factors (economic, political or cultural structures within a given society). There is ample evidence that social norms related to eating behaviors have a powerful effect on food choice [23,24,25]. To the best of our knowledge, no previous study has explored the relationship between social norms, the local food environment, and individual food purchasing behaviors. 

The Heart Healthy Hoods (HHH) [26] examines the urban environment (including the food environment) in relation to cardiovascular health in the city of Madrid. Within this project, we conducted a mixed-methods pilot study in Madrid, in a setting with high healthy food availability. Exploratory results indicated that studying residents’ perceptions might be a fundamental research step to understand how the local food environment changes over time [27]. 

The main objective of our study was to explore and describe the social norms relating to the local food environment and food purchasing behaviors, as described by residents and food traders using qualitative methods.

## 2. Materials and Methods

### 2.1. Methodological Approach

This research was guided by the social ecological model (SEM), which examines the interdependence between individuals, communities, and their environments [28]. SEM emphasizes the links and relationships between multiple levels and determinants of health, including family members, peers, school settings, food outlets, neighborhoods, media, culture, social norms, and the political system. This model also highlights the connections between individual or collective behaviors and the health resources existing in specific environmental settings, such as local food environments. This framework also helps understand the multifaceted and interactive effects of individual, social, and environmental factors influencing individual behaviors, such as dietary behaviors.

We chose a qualitative approach [29] to explore the perceptions of residents and food traders regarding their food environment. Qualitative research enables an understanding from the point of view of the actors involved, such as how residents and food traders view their neighborhood or what affects the way people buy food in their food environment.

### 2.2. Context and Study Setting

We conducted this study in a working-class neighborhood in the city of Madrid. We selected this area using the Median Neighborhood Index, a method that selects an area of a given population lying in the median of several variables: aging (% >65 years), low education (% with <8–9 years of education), segregation (% foreign-born), and urban form (population density per km^2^, used as a proxy). We used a spatial scan statistic to find clusters of median areas (low Median Neighborhood Index) with a maximum of 12 census sections, aiming to find a median neighborhood to explore the local food environment in a non-extreme setting within the city of Madrid [19]. The District of Ciudad Lineal comprises these median neighborhoods with median socio-economic characteristics. A more detailed description of the sociodemographics of the study area can be found in related articles [26,30].

### 2.3. Sampling and Participants

Our study sample included residents, neighborhood workers, and food traders of the neighborhood. We used a stratified purposive sampling strategy [31] to include informants that had been living or working in the area for a long time (more than 10 years) in order to maximize the information obtained, considering other variables of interest, such as sex, age, country of origin, and role in the neighborhood. This sampling strategy tries to reflect the diversity within a population rather than looking for statistical representativeness or generalizability [32]. The resulting sample consisted of 35 participants (*N* = 20 residents and neighborhood workers and *N* = 15 food traders).

Food store owners were not included in the sample as we prioritized food store workers who are those in daily contact with their customers and may have a better idea of the social norms and food purchasing behaviors in the area. Some store owners are owners of a chain of stores and never interact directly with customers.

We contacted and recruited participants by visiting neighborhood associations, neighborhood food stores, several educational and health centers, the Department of senior care services, and a public market. We explained the study aims to participants and invited them to participate. Once they agreed to participate, they completed written consent forms.

As shown in Table 1, participants’ ages ranged from 36 to 85, nine were females and eleven were males, two of them were migrants (one female and one male) and five were neighborhood workers. Table 1 also displays the characteristics of the food retailer types.

As for the food traders, all of them ran food stores that had been trading for at least one year. We classified these stores into three types, following previous fieldwork and published literature in Spain [19,33,34].

Type 1, small traditional stores, which are small establishments that may have been open for many years and are typically run by locals who are well-known in the neighborhood. These food stores are specialized food stores (e.g., fruit and vegetable stores, fishmongers, or butcheries). These stores are known in Spanish as *tiendas de toda la vida* (lifelong stores).

Type 2, convenience stores, typically run by immigrants, which have appeared in Spain relatively recently (the late 1990s and early 2000s). These stores may also be fruit and vegetable stores. These stores, such as stores selling Latin American products (e.g., special sauces or seeds) or Muslim products (e.g., halal products, spices, etc.), serve both autochthonous customers and immigrants. In this category, we also included stores typically run by Chinese, which have a much more diversified product offering, similar to convenience stores.

Type 3, small supermarkets which, on a small scale, allow the supply of all types of fresh and non-perishable food products and other non-food products for daily consumption.

### 2.4. Data Collection

We conducted semi-structured individual interviews (SSI) with key informants from the study area. In SSI, respondents can be perceived as the experiential experts on the subject and should, therefore, be allowed maximum opportunity to tell their own story [35]. The interviews were held between January 2015 and May 2016.

Three researchers conducted all 35 semi-structured interviews, which took place at different neighborhood facilities (primary health care center, school, food stores, and participants’ residences). Interviews lasted 40–50 min and were digitally recorded and transcribed verbatim for analysis.

Residents’ and workers’ interviews included general questions about the neighborhood (sociodemographic composition, geographic limits, main changes, and different uses) and more detailed questions about their food environment. All topic guide questions were based on previous literature and researchers’ experience and were specifically prepared for this study. Questions for food traders related to consumers’ purchasing patterns, changes in the food supply and demand, and the factors influencing the supply and demand for food (Table 2).

### 2.5. Data Analysis

Four researchers carried out the analysis of the interviews, following the quality criterion of investigator triangulation and incorporating an interpretative phenomenological analysis (IPA) perspective [36]: coding the data (trying to identify objects of concern, relationships, concepts, processes, etc.), looking for themes related with the objectives, and then connecting the themes with a pattern of meaning, revisiting previous text to revise initial coding, and allowing new categories to emerge inductively from the text. Data saturation [37] was reached for both populations (residents/workers and food traders).

This study was conducted in accordance with the Declaration of Helsinki, and it was approved by the Ethics Committee of the Universidad de Alcalá (CEI/HU/2017/09).

## 3. Results

Participants identified three main elements impacting their food purchasing behaviors, from individual elements to social context elements: (1) the relevance of interpersonal relationships, (2) the new fast pace of life, and (3) the economic crisis. These intermediate-level factors (social norms), such as interpersonal relationships and a new pace of life, are influencing residents’ food purchasing behaviors in a communal way (as a normative behavior within a social group). Both elements are influenced by the economic crisis, which is a macro-level factor.

### 3.1. Interpersonal Relationships: Affective Factors, Trust, and Tradition

Older residents reportedly bought most of their food in small traditional stores, their *tiendas de toda la vida* (lifelong stores). They highlighted the relationship of trust between them (customers) and the food traders, whom they have known for years as a social relationship which is handed down from generation to generation: “I go to La Elipa market and I already know the people… always have, of course, I’ve been going to the same butchery for 30 years.” (Resident, male, 63 years).

Personal attention, trust, and tradition are key elements. “The elderly people are more trusting. They still trust the product and the person who serves them while young people don’t care about trust but want speed, velocity, without thinking about whether you treat them well.” (Food trader, shop type 1).

Both residents and food traders spoke of affectionate relationships with type 1 stores. Residents regarded food traders as experts who provided them with plausible advice about the food. In turn, food traders explained how they achieved the loyalty of their customers through friendly treatment and trustworthy opinions: “Small stores survive because customers like going there and they know your name… I’ve got my customers. I know what her husband likes, what she likes, what her daughter likes, and so that trust is what we don’t get from the big supermarkets, and it’s what we’re looking for in the small stores.” (Food trader, store type 1).

“Because of the trust, the friendship, if someone who lives closer to a cheaper shop, but know this man, then he comes here every day to buy, it’s a matter of friendship, not of business, it’s about trust.” (Food trader, shop type 2).

### 3.2. New Pace of Life

Younger neighbors, or “new resident”, and working age residents, had different lifestyles, defined by a lack of time, which meant a “different way of shopping”. They preferred big supermarkets where products of all kinds (not just food) may be purchased faster.

“People don’t have time. The only time they have is on the weekend, and now, with the liberalization of store opening hours, what they do is take advantage of the weekends when they don’t work, Saturday afternoons or even Sundays, and they go to the supermarket and fill their trolley with all they can, and with all the supermarkets, the big supermarkets, and buy everything there” (Food trader, store type 1).

So, this new pace of life led to new behaviors: “Young people’s habits have changed, they’ve got very tight schedules, and a lot of young people don’t know how to cook a *pisto* or a *gazpacho*. Well, what are they going to do? … if they can buy it already made?” (Food trader, store type 1).

As for the most important factors regarding the use of supermarkets, they highlighted wide range of choices, more affordable prices, long opening hours, and the opportunity to be more time-efficient when shopping: “Yes, I go [to do the shopping], I get home at eight in the evening, I go to Ahorra Plaza or whatever, and I have to do the shopping. So, I think that it’s a lack of time that also makes people buy in those big places.” (Resident, female, 61 years).

Longer opening hours influenced customers numbers—above all, of those who work and have little time to shop in more limited commercial timetables: “Someone spends all day out working and then comes back home at 9 or 10. Where am I going to do the shopping, and what am I going to have for lunch or for dinner? That’s why I buy something simple here in the shop… you know the price here is a bit dear, but if nothing’s open at night, where do I do the shopping? Here.” (Food trader, store type 2).

### 3.3. Economic Crisis

Residents’ purchasing power largely decreased due to the 2008 economic and financial crisis. The economic crisis in Spain had as its main consequences: rising unemployment, precarious employment, and inequality in income distribution. This economic crisis in Spain started in 2008, fully developed in 2009 to 2010, and recovered in 2014 although important segments of the Spanish population have not fully recovered yet.

Due to this diminished purchasing power, people purchased less variety and less quantity: “Before, people bought much more in kilos, and now, they buy item by item. We don’t like leaving things in the freezer… So that’s how it goes. They buy a lot more by the piece, just enough so there are no leftovers.” (Food trader, shop type 1).

A decrease in dietary quality was associated with this slump in purchasing power: “I have hamburgers because my grandson has lunch with me, and as I’ve got… I don’t know, three or four euros; we either eat hamburgers, or we don’t eat at all.” (Resident, female, 53 years).

The main themes of the analysis are shown in Table 3.

## 4. Discussion

To our knowledge, this is one of the few qualitative studies focusing on why and how people purchase food in their neighborhood and on how social norms (relational, generational, and economic factors) shape food purchasing behaviors within the local food environment. This study adds both residents’ and food traders’ perspectives filling this important research gap.

Our results highlighted intermediate elements of the local food environment in relation to individual food purchasing behaviors, such as trust and tradition or the current lack of time to buy food and cook it at home. These intermediate elements, according to the social ecological model, are fundamental pieces for understanding how the local food environment relates to purchasing behaviors.

Food environment research has rarely used qualitative methodologies to understand consumers’ and food traders’ perceptions [38,39,40]. We consider it key to study residents’ perceptions as they are those who ultimately make, or do not make, the healthy food purchases. Food traders do also have a very important point of view; therefore, they should be included in these studies as key stakeholders [41,42]. Both are experts when studying the main changes occurring over time in their neighborhoods.

### 4.1. Interpersonal Relationship Elements

A key element in choosing where to buy was the retailer type because it related to the kind of relationship with the food trader. Older participants listed a series of variables which impacted on their choice for specialized food stores of type 1: the main variable is the friendly customer service, trust, and the food traders’ expertise. As previous studies have shown, customers appreciate an environment where they feel welcomed and accepted [43].

The explanatory force of these elements, as mentioned by the participants, is due to a mode of consumption “contaminated” by social and personal relationships within the neighborhood [44,45]. Thus, many of these small traditional food stores have closed, without others of a similar nature taking their place [46,47]. These results are consistent with previous research using quantitative methods [48], highlighting the progressive drop in the number of traditional stores, the appearance of discounters, the notable growth of supermarkets because of their diversified offering and near-cost prices, and the success of supermarkets rooted in their ability to open multiple points of sale.

### 4.2. New Pace of Life

Younger residents were much more pragmatic about their consumption habits than the elderly, tending, as they do, to have a different perception of time and to organize their purchases in a way that enables them to buy food fast (with no concern for affective relations or expert advice). This different form of consumption leads to the use of supermarkets and is related to other aspects of culinary behavior which set them apart from the elderly, such as their familiarity with cooking, or the lack of it. Young people either do not cook, or they consume oven-ready food, whereas the elderly revere cooking as a key element in the household and favor traditional dishes requiring greater preparation time. The expense of time is crucial, and these changes in attitudes and behaviors have also been found in previous publications [44,45,46]. This finding supports the idea that young people tend to consume more oven-ready food and therefore change to an unhealthy diet, as previous studies showed [49]. Previous research assessing the purchase and/or intake of unhealthy foods and beverages (e.g., ultra-processed foods or sugar-sweetened beverages) by purchase location have found that supermarkets contribute to both an increased consumption of these [50,51], as to an increased obesity prevalence [52].

Regarding the use of supermarkets, participants in our study highlighted the wide variety of products, the opportunity to save time, and the affordable prices that this type of food stores offers. This characterization has been also identified elsewhere [46,47,48,53], stressing the capacity of supermarkets to adapt to new paces of life. Opening hours were also key, given the accelerated pace of life and the extended working hours. Residents may choose food stores with a more flexible schedule that is compatible with theirs. These stores seem to be essential for time-challenged customers [54], a type of increasingly numerous consumers. These results also appeared in previous studies strengthening the importance of time constraints for food shopping and cooking as well as for the personal relationship with food access and highlighting that these factors were more determinant in low socioeconomic status areas [55,56].

The changes inherent from the transition from modernity to post-modernity include many dimensions of life: labor, personal relationships, gender equality, personal identity, and so forth [57,58]. In relation to this study, the current instability in most of the jobs and the inclusion of the female in the labor market have contributed to changes in the pace of life [59]. Flexibility is referred to as the opposite of stability in the job market. That is to say, flexible contracts are more unstable and with fluctuating schedules eventually influence the pace of life of the workers. Within these changes, we emphasize less leisure time, fewer differences in the gender roles, and a more pragmatic relation with the urban food environment, food purchasing behaviors, and eating [60,61].

### 4.3. Economic Crisis

The 2008 economic crisis frequently cropped up in many of the interviews that were conducted. This economic crisis in Spain had as its main consequences: rising unemployment, precarious employment, and inequality in income distribution [62]. It should be pointed out that when we conducted the interviews, the impact of the crisis had already been felt for more than eight years in Spain and its effects were being felt in many sectors of society [63].

This economic downturn had an effect on people’s diets. For example, there are grandparents taking care of their children (who moved back for economic reasons) and their grandchildren [64]. Therefore, grandparents are again cooking for the family and so the children and grandchildren changed their diets both in quantity and quality. These changes went hand in hand with new purchasing patterns, with smaller quantities, lesser variety, and only one day’s supply of food purchased at a time. Our study shows how the choice of where to shop and what to buy, in accordance with socio-economic factors, may be translated into a new social norm influencing the use of one kind of shop or another. For example, potential users of small traditional food stores may be forced to buy in other types of stores, such as supermarkets, since the latter may be cheaper. We would also stress the diminished quality and quantity of the food consumed by those interviewees affected by the crisis. These results are consistent with other studies [53,63,65]. As shown in other studies, greater affordability is associated with a significant increase in the purchase and consumption of healthier foods [66,67,68]. Therefore, considering the social context of food purchasing for low SES groups in order to reduce food-related health inequalities is warranted.

This study presents several limitations. First, the findings are subject to possible variations in the way data were collected, as food traders’ interviews were conducted during business hours and, in some cases, under time pressure. Second, in the case of interviews with Chinese traders, a translator was used; hence, some parts of the discourse may have been lost. Despite these data collection concerns, all participants’ responses were consistent with the questions in the interview topic guide and with the study goals. In order to minimize possible data analysis biases and to increase the validity of research findings we used investigator triangulation, both for coding and for results reviewing. Despite interpretation biases cannot be entirely ruled out, the findings from the different researchers came out with the same conclusions, so our confidence in the study findings increased. Finally, we were able to reach the theoretical data saturation.

There are many publications on the relationship between the food environment and health, but the dynamics of this relationship are highly complex and still poorly understood [69,70,71,72], even in research problematizing the obesogenic thesis [73]. The authors would like to stress the usefulness of adding qualitative methods and designs to urban public health nutrition research [74,75]. Although we regard it as essential to use objective measures of the local food environment, qualitative research may identify relationships and mechanisms impossible to explain in quantitative designs. At the local level, administrations could help maintain and open small traditional stores and engage local residents in participating in local food environment initiatives.

## 5. Conclusions

Residents and food traders identified new social norms relating to the local food environment, intra- and interpersonal, and macro-level factors that influence residents’ food purchasing behaviors. Older residents highlighted affective factors (trust and interpersonal relationships with vendors) as key elements in choosing where to do their grocery shopping. Younger residents highlighted convenience, a variety of products, and flexible opening hours as key determinants of their food purchasing behaviors. Finally, the impact of the economic crisis, influencing all inter- and intrapersonal food environment elements, was highlighted.

Understanding residents’ and food traders’ perceptions may provide important insights to develop culturally acceptable interventions that may ultimately improve residents’ diets and health.

## Figures and Tables

**Table 1:** Profile of neighborhood residents (*N* = 20) and food traders (*N* = 15) interviewed.

Neighborhood Residents (*N* = 20). M = Male; F = Female			Food Traders (*N* = 15). 1 = Small Traditional Stores; 2 = Immigrant Run Convenience Stores; 3 = Small Supermarkets
Sex	Age	Key Variable for Selection/Inclusion	Food Store Type and Description
M	48	Active worker resident	Type 1: Butchery
M	85	Retired resident	Type 1: Fishmonger
M	63	Active worker resident	Type 1: Fruit and vegetable store
M	62	Neighborhood association activist	Type 1: Poultry
M	42	Immigrant resident	Type 2: Latin American fruit and vegetable store
M	65	Retired resident	Type 2: Arab fruit and vegetable store
M	63	Local politician	Type 2: Latin American convenience store
M	36	Active worker resident	Type 2: Chinese-run convenience store
M	48	Active worker resident	Type 2: Chinese convenience store
F	45	Immigrant resident	Type 2: Chinese convenience store
F	69	Retired resident	Type 2: Chinese convenience store
F	83	Retired resident	Type 2: Chinese convenience store
F	58	Unemployed resident	Type 3: Small supermarket
F	54	Active worker resident	Type 3: Small supermarket
F	61	Active worker resident	Type 3: Small supermarket
F	41	Primary school teacher	
F	53	Primary health care doctor	
F	51	Head of Health Promotion Department	
F	57	Head manager of senior care centers	
F	38	Recreational and cultural activities technician	

**Table 2:** Neighborhood participants’ and food traders’ interview topic guide.

Neighborhood Participants	Food Traders
In your family, who is the person in charge of buying food? Where is the family food purchased? What elements of the neighborhood do you think influence purchase? Factors that influence the purchase (offers, types of food, variety, schedules, the location of the store, trust with the shopkeeper, quality, etc.). What is your perception about the food that you/your family eat at home? Do you believe that it is influenced by the neighborhood where you live? Do you think that the food environment changes that have taken place in the neighborhood have influenced your use of such stores? How? Why? Is it easy to eat healthily in your neighborhood? In that sense, have you seen any kind of change/evolution in the feeding practices of the people around you? Do you go out to eat/drink in your neighborhood?	Description of the business and its customers The time that the store has been present in the neighborhood. Best-selling products in the store. Types of customers in your store. The relationship between types of customers with product types.
	Changes in the supply and demand for food Food store changes over time (types of customers, ways to buy (weekly vs. daily)). Major food store changes that have occurred in the neighborhood.
	Factors influencing food supply and demand Factors that influence the purchase (offers, types of food, variety, schedules, place of the store, trust in the shopkeeper, quality, etc.). Influence on neighborhood residents’ patterns due to changes in food stores.

**Table 3:** Main results of residents’ perceptions.

Theme	Quote
Interpersonal relationships: affective factors, trust, and tradition.	“The small store also survives because customers like going and they know your name… I’ve got my customers. I know what her husband likes, what she likes, what her daughter likes, and so that trust is what we don’t get from the big supermarkets, and it’s what people are looking for in small shops.” (Food trader, store type 1). “I have been with them [store type 1] for over forty years … I tell them ‘I need a special milk … without lactose’ and they bring it to me. I have been buying there for 40 years” (Resident, female 69 years).
New pace of life, lack of time (look for variety, prices, and opening hours).	“…young people don’t care about trust, but want speed, velocity, without thinking about whether you treat them well, but I think they buy worse quality…” (Food trader, store type 1). “People buy everything, oven-ready food, in the supermarket, and at the market, only people who cook buy fresh things.” (Resident, female 54 years).
Reduced purchasing power relates to people purchasing a lesser variety of healthy foods and smaller quantities.	“Before, people bought much more in kilos, and now, they buy item by item. We don’t like leaving things in the freezer… So that’s how it goes, they buy a lot more by the piece, just enough so there’s no leftovers”. (Food trader, store type 1). “I have hamburgers because my grandson has lunch with me, and as I’ve got… I don’t know, three or four euros; we either eat hamburgers, or we don’t eat at all.” (Resident, female 53 years). “Now, instead of hake, they buy whiting, for instance… Yes, hake’s usually expensive, very expensive”. (Food trader, store type 1).
